# Iatrogenic Kaposi Sarcoma Precipitated by Anti-Tumor Necrosis Factor-Alpha (Anti-TNF-α) Therapy

**DOI:** 10.7759/cureus.13384

**Published:** 2021-02-16

**Authors:** Aathi Lakshmi Mariappan, Shreya Desai, Alberto Locante, Palak Desai, Javairia Quraishi

**Affiliations:** 1 Internal Medicine, Rosalind Franklin University of Medicine and Science, North Chicago, USA; 2 Pathology, AMITA Health Saints Mary and Elizabeth Medical Center, Chicago, USA; 3 Hematology and Oncology, AMITA Health Saints Mary and Elizabeth Medical Center, Chicago, USA

**Keywords:** kaposi sarcoma, adalimumab, tnf alpha inhibitor

## Abstract

Kaposi sarcoma (KS) is a vascular neoplasm caused by human gammaherpesvirus 8 (HHV-8). Four subtypes of KS are described: classic (Mediterranean), epidemic (acquired immunodeficiency syndrome (AIDS)-associated), endemic (sub-Saharan Africa), and iatrogenic. Iatrogenic KS due to tumor necrosis factor-alpha (TNF-α) inhibitor therapy is particularly rare. A 66-year-old female with a history of seropositive rheumatoid arthritis (RA) presented with a skin lesion on her right second toe. Diagnosed with RA four years prior, she failed to respond to methotrexate, hydroxychloroquine, and etanercept. As a result, she was started on adalimumab. Approximately two months into therapy, she presented to the emergency room with a dark brown skin lesion on her right second toe. She underwent excisional biopsy of the mass, which demonstrated a tumor composed of spindle cells forming slit-like spaces with extravasated red blood cells. The tumor was positive for cluster of differentiation 31 (CD31), CD34, and HHV-8 immunostains and negative for smooth muscle antibody (SMA) and desmin immunostains, consistent with Kaposi sarcoma. Human immunodeficiency virus (HIV) serology was negative. The patient was diagnosed with iatrogenic KS. Adalimumab was discontinued. The patient was started on alitretinoin and underwent adjuvant radiation therapy to minimize recurrence. TNF-α is a pro-inflammatory cytokine that has been implicated in many inflammatory diseases and in cell apoptosis. While anti-TNF-α agents have improved outcomes in many immune-mediated diseases, higher rates of infections and malignancy have also been reported. The incidence of KS with anti-TNF-α therapy remains a rare entity. Therefore, it is extremely important for patients receiving biologic agents, including TNF-α inhibitors, to have a close follow-up and receive routine skin evaluation for malignancy. Clinicians should have a high index of suspicion for KS in such non-HIV patients started on immunosuppressive agents.

## Introduction

Kaposi sarcoma (KS) is a vascular neoplasm caused by Kaposi sarcoma herpesvirus (KSHV), also known as human gammaherpesvirus 8 (HHV-8) [1]. Kaposi sarcoma is most frequently seen in regions with high KSHV seroprevalences such as sub-Saharan Africa and Mediterranean countries. Otherwise, it is classically known as an acquired immunodeficiency syndrome (AIDS)-defining illness. Four sub-types of KS are described: classic (Mediterranean), epidemic (AIDS-associated), endemic (sub-Saharan Africa), and iatrogenic [1-2]. The iatrogenic variant of KS is classically reported in organ transplant patients undergoing immunosuppressive therapy or those receiving long-term steroids [3]. However, over the past few decades, the use of biologic agents such as tumor necrosis factor-alpha (TNF-α) inhibitors has led to an increase in cases of KS. In this article, we report a case of iatrogenic KS caused by adalimumab with a brief review of other reported cases of KS in patients receiving TNF-α inhibitor therapy.

## Case presentation

**Figure 1:**
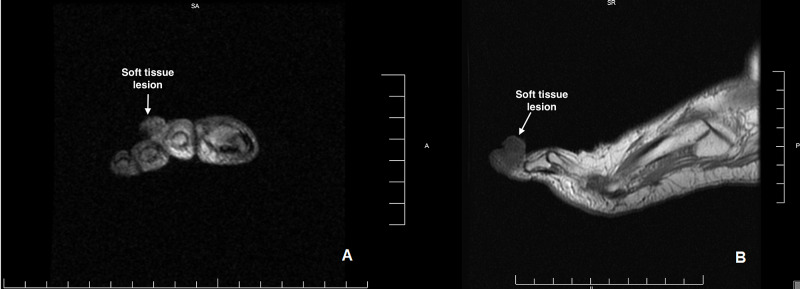
MRI of the soft tissue mass (arrow)

A 66-year-old female of middle eastern ethnicity with a history of seropositive rheumatoid arthritis (RA) was noted to have a skin lesion on her right second toe. Diagnosed with RA four years prior, she had failed to respond to methotrexate, hydroxychloroquine, and etanercept. As a result, she was started on adalimumab 40 mg every two weeks. Approximately two months into adalimumab therapy, the patient presented to the emergency room with a dark brown skin lesion on her right second toe. The lesion had been present for approximately three to four weeks, was slowly increasing in size, with intermittent tenderness and occasional bleeding. She did not report a history of diabetes or recent foot injuries. Physical exam showed a raised, brown lesion over the distal, dorsolateral second digit, approximately 1.4 cm in size, well-circumscribed from the surrounding tissue of the digit. A magnetic resonance imaging (MRI) scan revealed a 15.95 mm x 10.67 mm x 9.25 mm oval, circumscribed, exophytic, T1 intermediate intensity, T2 intermediate intensity mass in the subcutaneous soft tissues of the lateral second digit without the involvement of the underlying second extensor tendon or the second middle phalanx (Figures 1A-1B).

**Figure 2:**
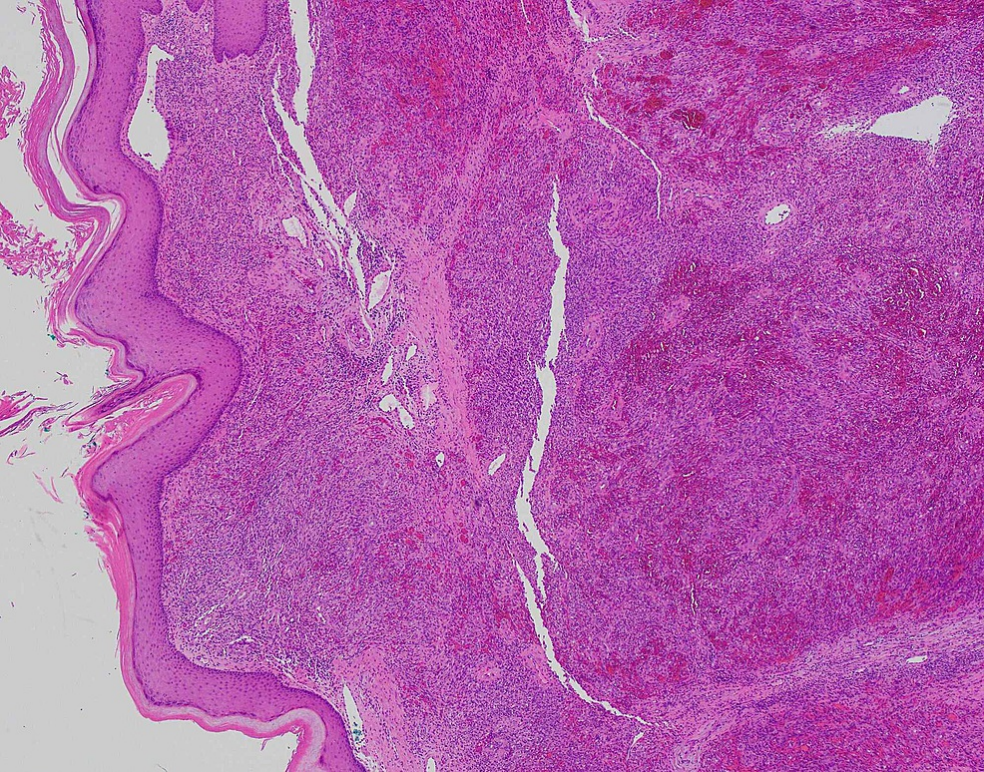
Hematoxylin and eosin stain at 40x magnification showing spindle cells forming slit-like spaces with extravasated red blood cells

**Figure 3:**
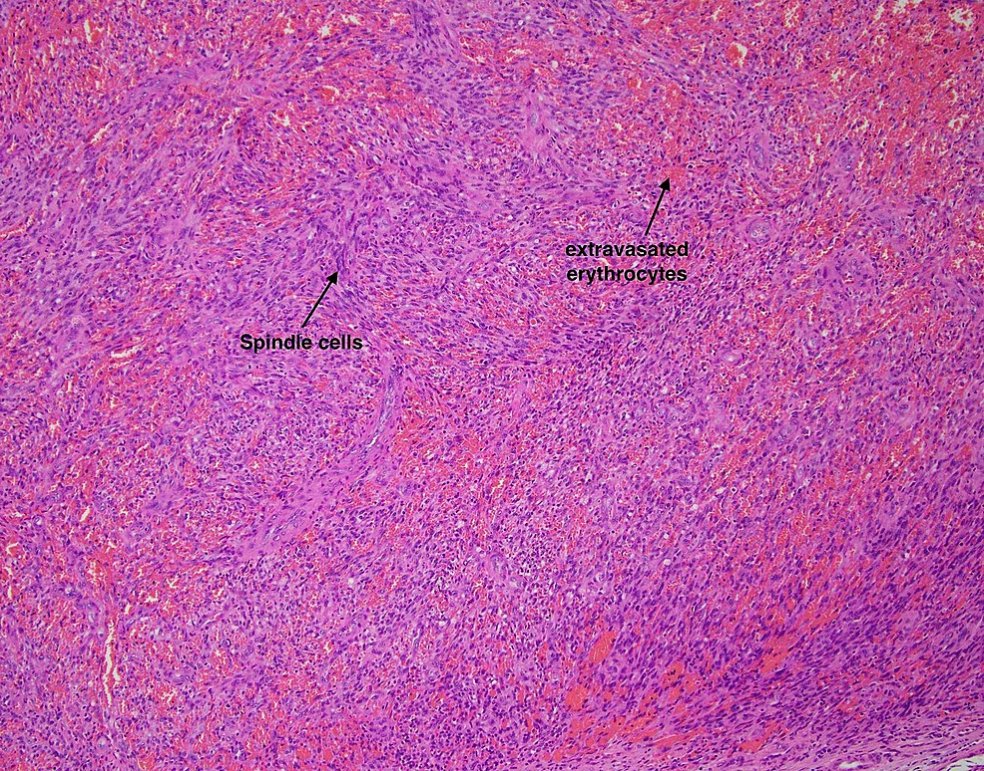
Hematoxylin and eosin stain at 100x magnification showing spindle cells forming slit-like spaces with extravasated red blood cells (arrows)

**Figure 4:**
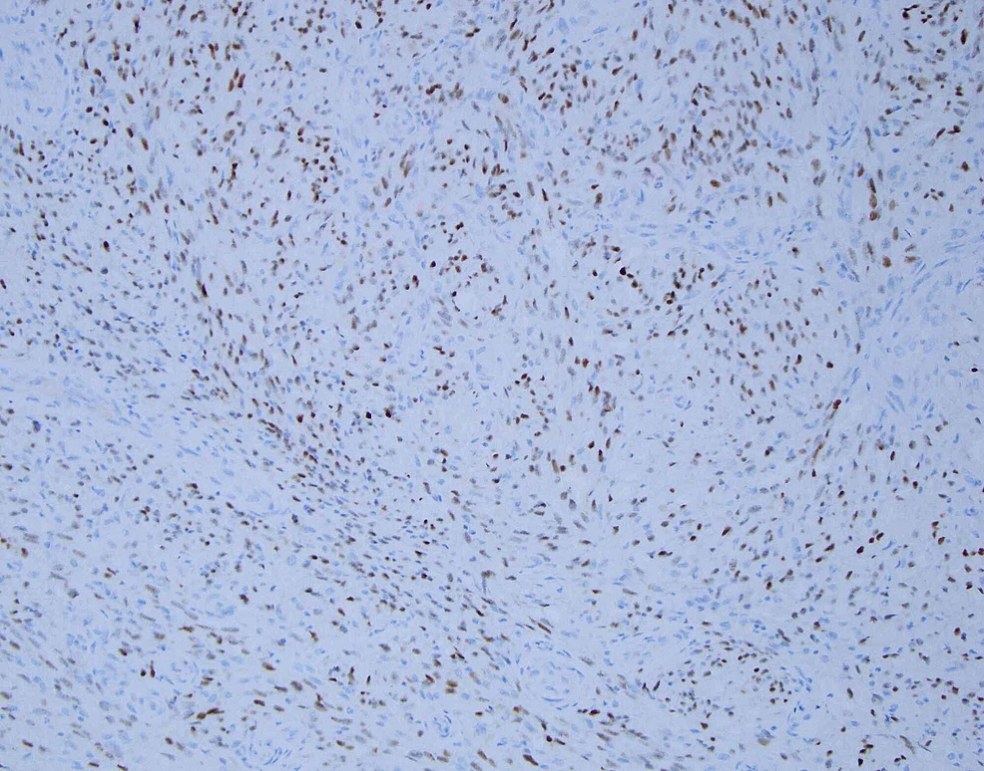
Positive HHV-8 immunostain at 200x magnification HHV-8: human gammaherpesvirus 8

She subsequently underwent an excisional biopsy, which microscopically demonstrated a tumor composed of spindle cells forming slit-like spaces with extravasated red blood cells (Figures 2-3). The tumor was positive for cluster of differentiation 31 (CD31), CD34, and HHV-8 immunostains and negative for smooth muscle antibody (SMA) and desmin immunostains, consistent with KS (Figure 4).

Human immunodeficiency virus (HIV) serology was negative. Adalimumab was discontinued. The patient was started on alitretinoin and underwent adjuvant radiation therapy to minimize recurrence.

## Discussion

TNF-α is a pro-inflammatory cytokine that has been implicated in numerous inflammatory diseases and in cell apoptosis [4]. Treatment with anti-TNF-α agents thus has improved outcomes in many immune-mediated diseases, including rheumatoid arthritis (RA), psoriasis, Crohn’s disease, etc. At the same time, higher rates of infections and malignancy have also been reported. A meta-analysis of 20,648 patients with RA found a higher risk of non-melanoma skin cancer (NMSC) in patients receiving TNF-α antagonists as compared to those treated with non-biologic disease-modifying antirheumatic drugs (DMARDs) with a hazard ratio of 1.42 (95% CI 1.24-1.63) [5]. Additionally, the duration of therapy with anti-TNF-α agents was not found to be a notable risk factor for the development of NMSC [5-6]. 

To our knowledge, five cases of KS were identified with the use of infliximab, two cases with adalimumab, one with golimumab, and one with certolizumab pegol [7-15]. In most of the cases, KS was notably localized to the skin. All patients tested negative for HIV. The onset of KS was noted weeks to months following initiation of treatment with an anti-TNF-α therapy agent. Due to the rarity of the disease in this patient population, diagnosis can often be missed or delayed. Therefore, it is extremely important for patients receiving biologic agents, including anti-TNF-α therapy, to have a close follow-up and receive routine skin evaluation for malignancy. Additionally, clinicians should have a high index of suspicion for KS in such non-HIV patients started on immunosuppressive agents.

## Conclusions

Rare reports of iatrogenic Kaposi sarcoma in non-HIV patients receiving anti-TNF-α therapy have been identified. Patients receiving TNF-α inhibitors should undergo routine skin evaluation for malignancy.
